# Cardiac Tamponade and Autoimmune Hemolytic Anemia Associated With Systemic Lupus Erythematosus: A Case Report and Literature Review

**DOI:** 10.7759/cureus.106445

**Published:** 2026-04-04

**Authors:** Motaz Almahmood, Hatem Ahmed, Ayah Al Qaryoute, Ibrahim H Inanc, Muhammad Ali Tariq

**Affiliations:** 1 Internal Medicine, Tower Health Medical Group, Phoenixville, USA; 2 Physiology, Appalachian State University, Boone, USA; 3 Cardiology, Kırıkkale Yüksek İhtisas Hastanesi, Kırıkkale, TUR

**Keywords:** autoimmune hemolytic anemia (aiha), cardiac tamponade, direct antiglobulin test (dat), emergency pericardiocentesis, late-onset lupus, pericardial effusion, serositis, systemic lupus erythematosus, warm aiha, warm autoimmune hemolysis

## Abstract

Systemic lupus erythematosus can present with varied clinical features, and life-threatening serositis or autoimmune hemolysis may precede more typical findings. We report a 56-year-old woman who presented with progressive dyspnea, pleuritic chest discomfort, hypotension, and tachycardia and was found to have a large pericardial effusion with tamponade physiology requiring urgent pericardiocentesis. During hospitalization, worsening anemia with positive direct antiglobulin testing, peripheral spherocytes, reticulocytosis, hyperbilirubinemia, low haptoglobin, and splenomegaly supported the diagnosis of warm autoimmune hemolytic anemia. Infectious, malignant, and other causes of inflammatory pericardial effusion were excluded. The overall presentation, including serositis, autoimmune hemolysis, and positive lupus serologies, supported the diagnosis of late-onset systemic lupus erythematosus. She was treated with pericardiocentesis, corticosteroids, transfusion support, intravenous immunoglobulin, rituximab, and later hydroxychloroquine, with resolution of the pericardial effusion and improvement in anemia. This case emphasizes that systemic lupus erythematosus should be considered in adults with otherwise unexplained cardiac tamponade accompanied by hemolysis, even when classic mucocutaneous or musculoskeletal manifestations are absent at presentation.

## Introduction

Systemic lupus erythematosus (SLE) is a chronic autoimmune disease with protean clinical manifestations and may involve nearly any organ system [1,2]. Serositis is a recognized feature within the 2019 European Alliance of Associations for Rheumatology/American College of Rheumatology classification criteria, and pericarditis is among the best-described cardiac manifestations of lupus [1,3]. Pericardial involvement is relatively common in SLE, with pericarditis reported in approximately 11-54% of patients, whereas progression to hemodynamically significant cardiac tamponade is uncommon and may represent the initial manifestation of disease in only rare cases [3-6].

Hematologic abnormalities are also central to lupus recognition. Autoimmune hemolytic anemia (AIHA) is a weighted hematologic feature in the current classification criteria, and warm AIHA may occur secondary to autoimmune disease, including SLE [1,7]. Although warm AIHA has been described as an initial presentation of SLE, this remains an unusual pattern of disease onset [7,8]. Late-onset SLE may further complicate recognition because initial manifestations may be atypical and the diagnosis may not be immediately apparent at presentation [9].

The concurrent presentation of cardiac tamponade and warm AIHA at the initial diagnosis of SLE appears to be distinctly uncommon in adults, with only rare case reports describing this combination [10-12]. Recent reports continue to show that cardiac tamponade may herald SLE even in the absence of concurrent AIHA, underscoring the unusual clinical significance of their coexistence rather than either manifestation in isolation [10-13]. We present a case of late-onset SLE in a 56-year-old woman whose initial presentation was dominated by life-threatening cardiac tamponade and direct antiglobulin test (DAT)-positive warm AIHA, highlighting the need to consider lupus in adults with otherwise unexplained pericardial tamponade accompanied by autoimmune hemolysis.

## Case presentation

A 56-year-old woman with a history of Hashimoto thyroiditis with hypothyroidism and prior cholecystectomy presented with one week of progressively worsening shortness of breath. Initially, dyspnea occurred only with exertion, but it progressed to dyspnea with minimal activity and during conversation. She also reported pleuritic chest discomfort. She denied fever, chills, upper respiratory symptoms, rash, oral ulcers, photosensitivity, arthralgia, weight loss, or other infectious symptoms.

On presentation, she was hypotensive and tachycardic, with blood pressure as low as 81/24 mmHg, heart rate 120-126 beats/min, respiratory rate up to 30 breaths/min, and oxygen saturation of 98% on room air. She received a 1 L bolus of normal saline. Initial laboratory findings and selected infectious studies are summarized in Table 1. Renal function was preserved (creatinine 0.81 mg/dL, blood urea nitrogen 14 mg/dL), and urinalysis was negative for protein.

**Table 1:** Laboratory test results at the time of admission and selected infectious studies. Selected infectious studies are shown because hypotension at presentation prompted evaluation for systemic infection and alternative causes of inflammatory pericardial effusion. Ag: antigen; Ab: antibody; HBsAg: hepatitis B surface antigen; anti-HBs: antibody to hepatitis B surface antigen; anti-HBc: antibody to hepatitis B core antigen

Parameters	Results	Reference range
White blood cell count	8.6×10³/µL	4.0-10.8×10³/µL
Hemoglobin	7.5 g/dL	12.0-16.0 g/dL
Mean corpuscular volume	85 fL	80-99 fL
Platelet count	403×10³/µL	150-450×10³/µL
Blood urea nitrogen	14 mg/dL	9-23 mg/dL
Creatinine	0.81 mg/dL	0.55-1.02 mg/dL
Urinalysis	Negative for protein	No proteinuria
Lactate	3.0 mmol/L	0.6-1.4 mmol/L
Total bilirubin	2.0 mg/dL	0.3-1.2 mg/dL
C-reactive protein	18.3 mg/dL	<0.5 mg/dL
Erythrocyte sedimentation rate	37 mm/h	0-30 mm/h
Selected infectious studies		
Blood cultures	Negative	
Urine culture	Negative	
HIV antibodies	Negative	
HBsAg	Negative	
Anti-HBs	Negative	
Anti-HBc	Negative	
Hepatitis C Ab	Negative	

**Figure 1:**
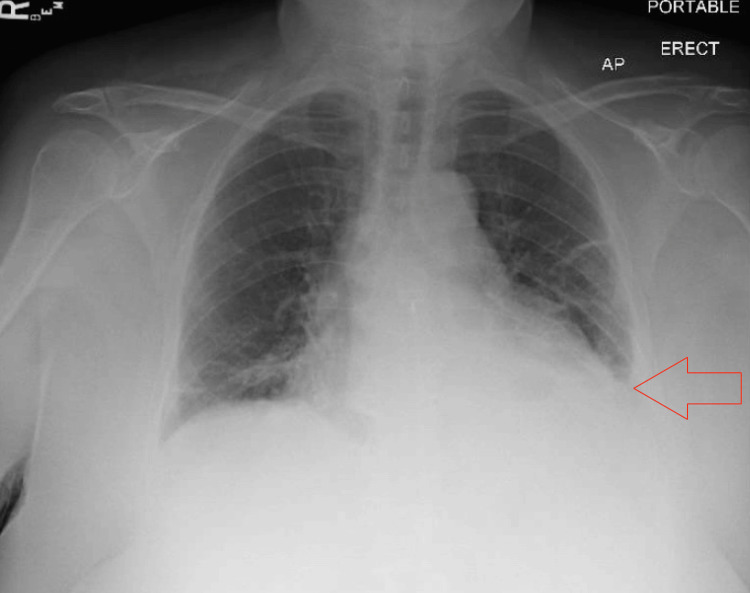
Portable chest radiograph demonstrating a small left pleural effusion (red arrow) and bilateral linear opacities consistent with atelectatic change.

**Figure 2:**
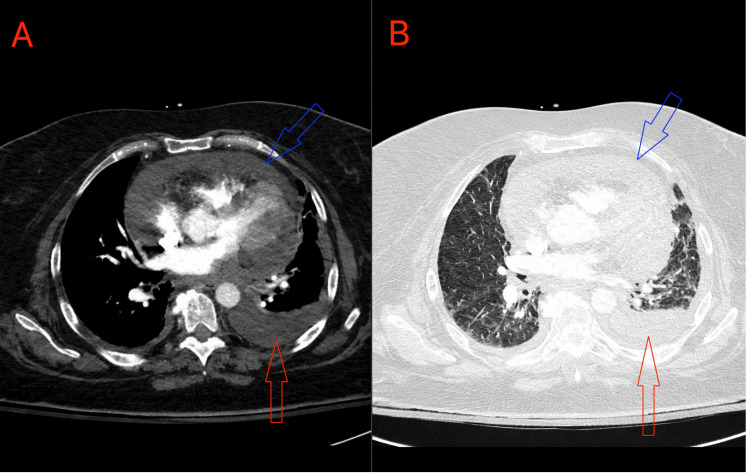
Computed tomography pulmonary angiography. (A) Mediastinal window and (B) lung window showing a large pericardial effusion (blue arrows) and bilateral pleural effusions, greater on the left (red arrows), without evidence of pulmonary embolism.

Portable chest radiography demonstrated a small left pleural effusion and bilateral linear opacities consistent with atelectatic change (Figure 1). Computed tomography pulmonary angiography showed no pulmonary embolism but revealed a large pericardial effusion and bilateral pleural effusions, greater on the left (Figures 2A, 2B).

**Figure 3:**
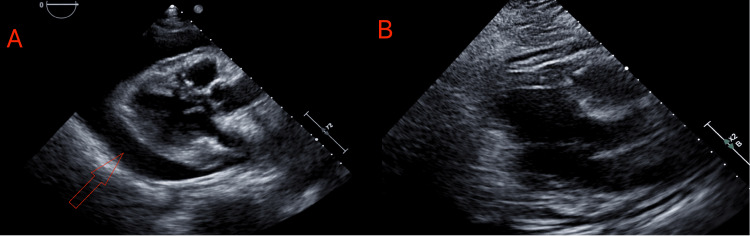
Transthoracic echocardiography before (A) and after (B) pericardiocentesis. (A) A large pericardial effusion before drainage and (B) marked reduction in the effusion after pericardiocentesis.

Bedside transthoracic echocardiography demonstrated a large pericardial effusion with tamponade physiology, and she underwent emergent echo- and fluoroscopy-guided pericardiocentesis with removal of 450 mL of serosanguineous pericardial fluid, followed by placement of a pericardial drain (Figure 3A). Follow-up transthoracic echocardiography showed marked reduction of the effusion after drainage, with only a minimal residual collection (Figure 3B). Pericardial fluid analysis supported the diagnosis of an inflammatory effusion (Table 2).

**Table 2:** Pericardial fluid analysis with microbiologic and cytologic evaluation.

Test	Result
Appearance	Serosanguineous
Protein	5.6 g/dL
pH	7.1
Nucleated cell count	27,066 cells/µL
Differential	90% segmented neutrophils
Bacterial cultures	Negative
Acid-fast bacilli culture	Negative
Fungal culture	Negative
Cytology	Negative

**Figure 4:**
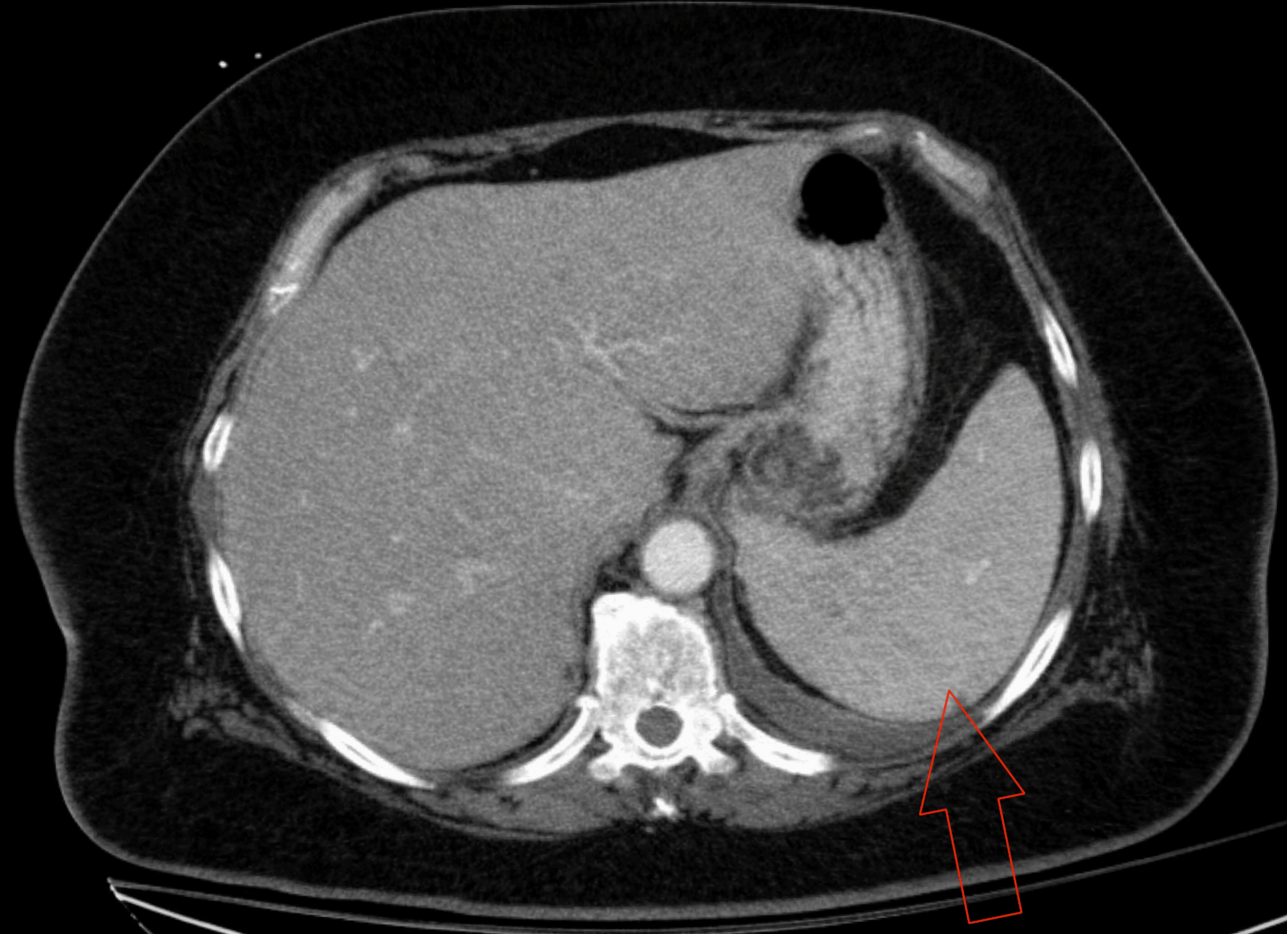
Computed tomography of the abdomen and pelvis demonstrating splenomegaly (red arrow).

**Figure 5:**
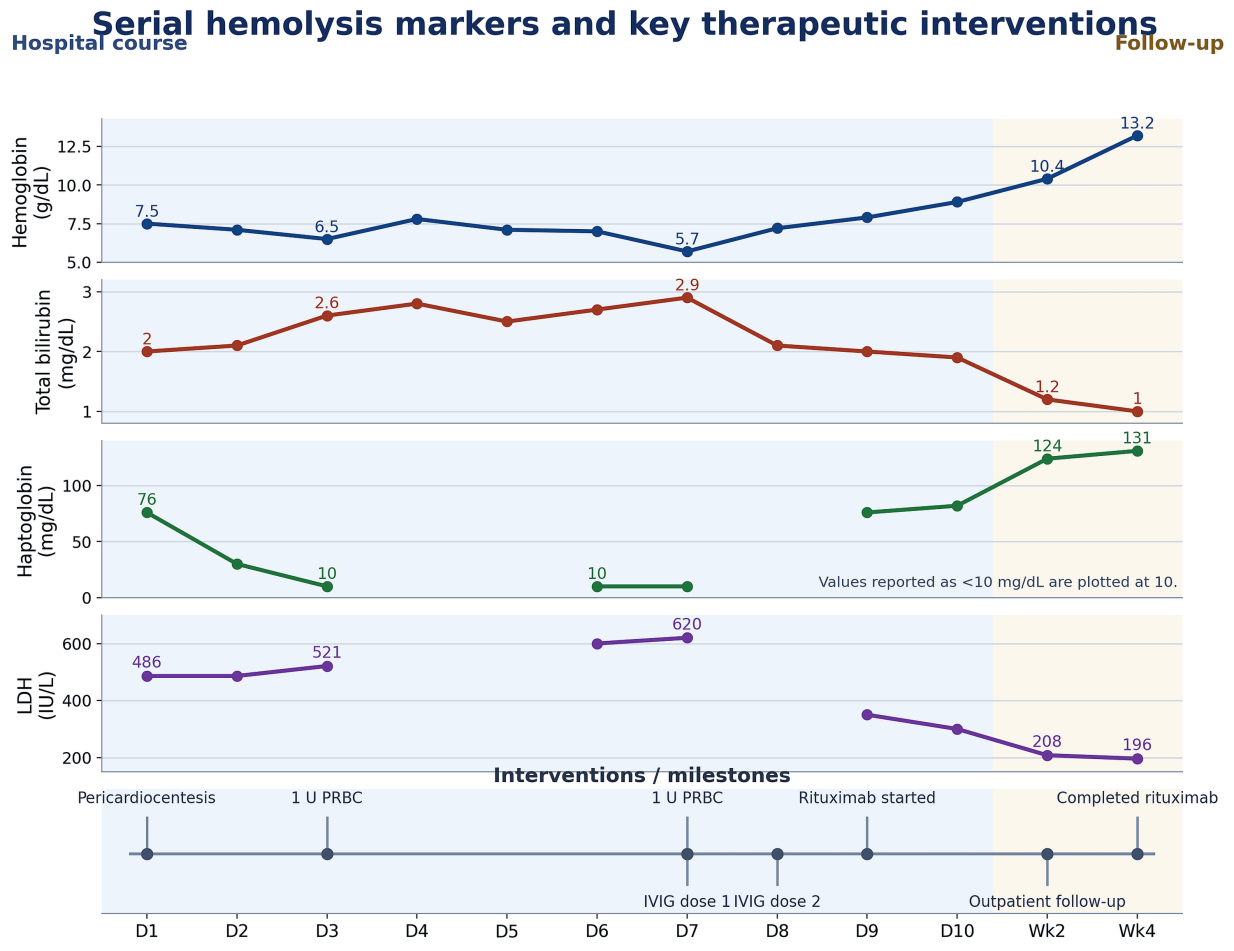
Serial hemolysis markers (including total bilirubin) and key therapeutic interventions from admission through four-week follow-up. IVIG: intravenous immunoglobulin; LDH: lactate dehydrogenase; PRBC: packed red blood cells; U: units

Although the pericardial fluid was neutrophil-predominant, bacterial, fungal, and acid-fast bacilli studies were negative, and cytology did not show malignant cells; these findings argue against infectious and malignant etiologies. Subsequent autoimmune evaluation demonstrated positive lupus serologies, which, together with concurrent hemolysis, supported systemic lupus erythematosus (SLE) as the unifying diagnosis. Hemolysis was present on admission and became more clinically apparent during hospitalization, with worsening anemia despite supportive care. Hemolysis evaluation was notable for elevated lactate dehydrogenase, indirect-predominant hyperbilirubinemia, low haptoglobin, and reticulocytosis. Peripheral blood smear showed spherocytes. Computed tomography of the abdomen and pelvis demonstrated splenomegaly (Figure 4). The serial hemolysis course and major interventions are summarized in Figure 5, and the hemolysis and autoimmune workup are summarized in Table 3. Direct antiglobulin testing was positive by polyspecific direct antiglobulin test (DAT), with monospecific reactivity for both IgG and C3; in the overall clinical context, the hemolysis was most consistent with warm autoimmune hemolytic anemia.

**Table 3:** Hemolysis and autoimmune workup. DAT: direct antiglobulin test; LDH: lactate dehydrogenase; ARC: absolute reticulocyte count; ANA: antinuclear antibody; AIHA: autoimmune hemolytic anemia; SLE: systemic lupus erythematosus

Test	Results	Comment/normal references
Hemolysis workup		
DAT	Positive (polyspecific); monospecific DAT positive for IgG and C3	Overall, most consistent with warm AIHA in the clinical context
LDH	486 U/L on admission; peak 620 U/L during hospitalization	120-246 U/L
Haptoglobin	<10 mg/dL during hospitalization	30-200 mg/dL
Reticulocyte count	4.5%	0.5-2.0%
ARC	159.8×10⁹/L	30.4-110.9×10⁹/L
Peripheral blood smear	Spherocytes	Supports hemolysis/warm AIHA
Total bilirubin	2.0 mg/dL on admission; peak 2.9 mg/dL	0.3-1.2 mg/dL
Indirect bilirubin	1.8 mg/dL	0.2-0.8 mg/dL
Direct bilirubin	0.2 mg/dL on admission	Normal
Bilirubin fractionation	Indirect-predominant hyperbilirubinemia	Supports hemolysis
Autoimmune workup		
ANA	Positive, homogeneous pattern, titer 1:320	Strongly positive autoimmune serology
Anti-double-stranded DNA antibody	Positive	-
Anti-Smith antibody	Negative	-
C3	121 mg/dL	83-193 mg/dL
C4	17 mg/dL	15-57 mg/dL

Clinically, her dyspnea and hemodynamic instability improved promptly after pericardiocentesis. She received one unit of packed red blood cells on hospital day three and an additional unit on hospital day seven. Initial treatment included prednisone 1 mg/kg/day. Because hemolysis and anemia persisted, intravenous immunoglobulin was initiated on hospital day seven at 1 g/kg/day for two doses, followed by rituximab 375 mg/m² administered intravenously once weekly for four weeks. Given the combination of pericardial serositis with cardiac tamponade, polyspecific direct antiglobulin test-positive autoimmune hemolytic anemia, positive ANA and anti-dsDNA serologies, and arthralgias that developed on follow-up after being absent at initial presentation, hydroxychloroquine was started in the outpatient setting after rheumatology evaluation, once the diagnosis of SLE had become clearer and the immediate inpatient priorities of tamponade stabilization and active hemolysis management had resolved.

Serial echocardiograms demonstrated progressive resolution of the pericardial effusion, with only trace residual fluid and no recurrent tamponade. Her hemoglobin improved to 10.4 g/dL at two weeks after discharge and to 13.2 g/dL at four weeks after discharge. She completed four weekly doses of rituximab and underwent a gradual prednisone taper on discharge. At follow-up, she remained under the care of hematology, cardiology, and rheumatology for systemic lupus erythematosus-associated autoimmune hemolytic anemia and lupus-related pericardial disease.

## Discussion

This case is notable because the patient presented with a rare combination of life-threatening pericardial tamponade and DAT-positive warm AIHA before classic lupus features were fully expressed. In SLE, pericarditis is relatively common, whereas hemodynamically significant tamponade is uncommon and may mimic infection, malignancy, or other inflammatory causes of pericardial effusion [3-6]. In parallel, AIHA is a recognized hematologic manifestation of SLE and often occurs near disease onset, but it is still far less common than anemia of chronic inflammation or iron deficiency [7,8]. The diagnostic challenge in this patient was therefore not whether either manifestation can occur in lupus, but that both appeared together and dominated the initial presentation in an older adult [9].

The key distinguishing feature of the present case is the concurrence of both processes at the initial presentation of SLE. A focused search of PubMed and Google Scholar was performed using combinations of the terms “systemic lupus erythematosus,” “cardiac tamponade,” “pericardial effusion,” “autoimmune hemolytic anemia,” and “hemolytic anemia.” Three previously reported adult cases describing the initial presentation of SLE with both cardiac tamponade and hemolytic anemia or AIHA were identified and are summarized in Table 4 alongside the present case. Abraham III et al. described a 44-year-old African-American woman whose presenting illness included cardiac tamponade, with hemolytic anemia among the presenting SLE features [10]. Chourabi et al. reported a 22-year-old woman in whom cardiac tamponade led to the diagnosis of SLE, with hemolytic anemia among the accompanying diagnostic features [11]. Dubey et al. described a 23-year-old woman with acute pericarditis, cardiac tamponade, and AIHA as presenting manifestations of SLE [12]. Recent adult case reports also confirm that cardiac tamponade at SLE presentation continues to be described even without concurrent AIHA [13]. Accordingly, the novelty of the present case lies not in tamponade alone or AIHA alone, but in the rare convergence of these two serious manifestations at the time SLE first became clinically apparent.

**Table 4:** Published adult case reports of initial systemic lupus erythematosus presenting with both cardiac tamponade and hemolytic anemia or autoimmune hemolytic anemia, compared with the present case. SLE: systemic lupus erythematosus; ANA: antinuclear antibody; AIHA: autoimmune hemolytic anemia; IVIG: intravenous immunoglobulin; DAT: direct antiglobulin test

Studies	Patient/gender	Initial presentation	Hemolysis	Key treatment/outcome
Abraham III et al. (2006) [10]	44/female	Worsening dyspnea and chest discomfort; cardiac tamponade preceded SLE diagnosis.	Hemolytic anemia was part of the presenting SLE criteria.	Case report established adult inaugural coexistence of tamponade and hemolytic anemia.
Chourabi et al. (2020) [11]	22/female	Dyspnea, chest pain, and cardiac tamponade are the initial presentation of SLE.	Hemolytic anemia was among the diagnostic features, along with serositis, arthralgia, ANA/anti-Sm positivity, and low complement.	Treated with IV methylprednisolone, oral prednisone, and hydroxychloroquine; complete resolution of the effusion without recurrence.
Dubey et al. (2023) [12]	23/female	Breathlessness, chest pain, acute pericarditis, and cardiac tamponade at initial SLE diagnosis.	Explicit AIHA reported.	Pericardiocentesis plus methylprednisolone and hydroxychloroquine; early follow-up showed marked clinical improvement and no active pericarditis.
Present case	56/female	Progressive dyspnea, hypotension, and large inflammatory pericardial effusion with tamponade physiology.	DAT-positive warm AIHA with reticulocytosis, spherocytes, hyperbilirubinemia, low haptoglobin, and splenomegaly.	Pericardiocentesis, corticosteroids, IVIG, rituximab, and hydroxychloroquine; no recurrent tamponade and progressive hemoglobin recovery.

In the present case, the inflammatory, serosanguineous pericardial fluid, negative microbiologic and cytologic studies, positive ANA and anti-dsDNA serologies, polyspecific DAT positivity, peripheral spherocytes, low haptoglobin, hyperbilirubinemia, reticulocytosis, and splenomegaly collectively supported a diagnosis of lupus-associated warm AIHA with serositis-related tamponade. This pattern is clinically relevant because attribution can be difficult when the first presentation is cardiopulmonary instability rather than typical mucocutaneous or musculoskeletal disease. Notably, complement levels were within the reference range, which does not exclude SLE, particularly in older adults or in cases where diagnosis rests on the overall clinicoserologic pattern rather than a single laboratory profile. Although classification criteria do not replace clinical judgment, the overall presentation was also compatible with the 2019 EULAR/ACR SLE classification framework, with ANA as the entry criterion and weighted features including serositis, autoimmune hemolysis, and anti-dsDNA positivity [1].

The patient’s age is also clinically relevant. SLE beginning after the age of 50 years is often described as late-onset SLE and may present with fewer classic mucocutaneous manifestations, less obvious renal disease, and a greater prominence of serosal or cardiopulmonary findings, which can delay diagnosis [9]. In this context, the absence of typical lupus features at presentation does not argue against SLE and may instead reflect the atypical phenotype seen in older adults.

Management required simultaneous treatment of the hemodynamic emergency and the underlying immune process. Urgent pericardiocentesis was essential for tamponade relief, while corticosteroids targeted both lupus serositis and immune hemolysis [6,7]. Because hemolysis persisted despite initial corticosteroid therapy, escalation to intravenous immunoglobulin and rituximab was clinically reasonable. Rituximab is an established option for steroid-refractory or steroid-dependent warm AIHA and is increasingly used when rapid steroid-sparing control is needed [14,15]. The favorable short-term course in this patient, with no recurrent tamponade and progressive hematologic recovery, underscores the value of prompt drainage, early autoimmune evaluation, and coordinated cardiology-rheumatology-hematology follow-up.

## Conclusions

This case illustrates that systemic lupus erythematosus in an older adult may initially present with the uncommon combination of life-threatening cardiac tamponade and autoimmune hemolytic anemia rather than with classic mucocutaneous or musculoskeletal features. In adults with otherwise unexplained pericardial effusion or tamponade accompanied by hemolysis, early evaluation for autoimmune disease may accelerate diagnosis and guide timely therapy. Prompt pericardial drainage together with appropriate immunosuppressive treatment can lead to favorable short-term outcomes.
